# Recurrent PRES in a Patient With Cyclic Vomiting Syndrome

**DOI:** 10.1155/crra/3914162

**Published:** 2026-06-26

**Authors:** Arman Mahmood, Allen Mao, Ibrahim Sacit Tuna

**Affiliations:** ^1^ University of Florida College of Medicine, Gainesville, Florida, USA, ufl.edu; ^2^ University of Florida Shands Hospital, Gainesville, Florida, USA; ^3^ University of Florida Department of Radiology, Gainesville, Florida, USA

## Abstract

Cyclic vomiting syndrome (CVS) is a functional gastrointestinal disorder defined by recurrent, stereotyped episodes of severe nausea and vomiting separated by symptom‐free intervals, and it is frequently accompanied by autonomic dysregulation and, in some patients, hypertension. Posterior reversible encephalopathy syndrome (PRES) is a clinicoradiologic syndrome commonly linked to acute hypertension and characterized by vasogenic edema on neuroimaging. We describe a 16‐year‐old female with a long‐standing, criteria‐based diagnosis of CVS and chronic, recurrent hypertension who developed MRI‐confirmed PRES during a period of sustained postoperative hypertension. She had a prior MRI‐confirmed episode of PRES in 2012, with documented radiologic reversibility on follow‐up imaging. Neuroimaging during the index admission demonstrated vasogenic edema in the parieto‐occipital lobes and cerebellar vermis. Secondary causes of hypertension, including pheochromocytoma, primary aldosteronism, thyroid disease, renal and renovascular disease, and Cushing syndrome, were systematically excluded, and the episode was considered most likely related to her hypertension. We discuss CVS‐related autonomic dysregulation as a possible contributor, while recognizing hypertension as the immediate driver of PRES in this case. Prompt antihypertensive therapy and seizure prophylaxis were followed by clinical and radiologic improvement. This case highlights the value of recognizing recurrent hypertensive PRES in adolescents with CVS and of carefully excluding alternative etiologies.

## 1. Introduction

Cyclic vomiting syndrome (CVS) is a condition defined by sudden, recurrent episodes of severe nausea and relentless vomiting, with episodes typically lasting from several hours to a few days in a predictable, stereotypical pattern (1). Its diagnosis is clinical and is anchored by the Rome IV criteria, which require stereotyped, self‐limited episodes of vomiting separated by intervals of return to baseline health, together with reasonable exclusion of structural, metabolic, and alternative gastrointestinal causes (2). CVS is frequently accompanied by autonomic dysregulation, predominantly sympathetic, and by hypertension in a subset of patients (3). A described phenotype, the Sato variant, additionally couples periodic vomiting with hypertension and neuroendocrine findings including catecholamine discharge (4). It is known that acute hypertensive episodes are a risk factor for posterior reversible encephalopathy syndrome (PRES). PRES is a neurological disorder of acute or subacute onset, characterized by a wide range of neurological symptoms such as headache, seizures, visual disturbances, confusion, and focal deficits, often associated with elevated blood pressure and distinctive neuroimaging findings including vasogenic edema (5).

We present a case of recurrent, predominantly hypertensive PRES in an adolescent with long‐standing CVS, including two imaging‐confirmed episodes over her lifetime, and discuss hypertension and CVS‐related autonomic dysregulation as the most plausible contributors.

## 2. Case Presentation

Our patient is a 16‐year‐old female with a complex history including CVS with associated hypertension, congenital esophageal achalasia (status post Heller myotomy and partial fundoplication), eosinophilic esophagitis, gastroesophageal reflux disease (GERD), a 22q11.2 duplication, an inflammatory myofibroblastic tumor of the right chest (status postresection), and feeding difficulty status post multiple gastrostomy and gastrojejunostomy procedures, who was admitted for excision and closure of a gastrocutaneous fistula. Her cyclic vomiting had been diagnosed in early childhood, with the first episode at 4 years of age, and was followed longitudinally by pediatric gastroenterology, nephrology, and behavioral health across several institutions. Her episodes were stereotyped, lasted approximately 24–36 h, recurred at least 1 week apart with return to baseline health between events, and met Rome IV criteria. Although she carried structural diagnoses of achalasia and eosinophilic esophagitis, her cyclic, self‐limited vomiting episodes were considered a distinct CVS phenotype after extensive multi‐institutional evaluation, including serial upper endoscopy with biopsy, esophageal manometry, and diagnostic laparoscopy, that did not identify an alternative explanation for these episodes (2). At the time of admission, her blood pressure was normotensive at 133/65 mmHg. Over the next few days, she underwent additional surgeries due to wound reopening; following the last surgery, her blood pressure rose to 160/126 mmHg and remained elevated for several days.

Two days after her final procedure, the patient developed vision loss, abnormal movements, and an inability to count her fingers. At the time of assessment, her blood pressure was hypertensive at 144/103 mmHg. She experienced a seizure‐like event, prompting a referral to pediatric neurology, who documented generalized shaking and reduced responsiveness. Although she had gastrointestinal symptoms during the admission, these occurred in the postoperative setting and were not clearly documented as a stereotyped CVS episode. Her blood pressure remained elevated postoperatively, and the neurologic episode was considered most likely related to hypertension. She was not receiving medications commonly associated with PRES, aside from short‐course dexamethasone administered on the day of and the day after her initial procedure. The patient had experienced a similar event previously. In 2012, she developed an episode confirmed as PRES on MRI: An initial study (9/8/2012) demonstrated new ill‐defined T2/FLAIR hyperintensities in the subcortical white matter of the right posterior frontal and bilateral parietal and parieto‐occipital regions, reported as most consistent with PRES, and a follow‐up study (9/28/2012) showed interval resolution of these changes, confirming reversibility. She had additional hospitalizations for vomiting with hypertension in 2018, 2019, 2022, and 2023; these were not confirmed as PRES on neuroimaging. Relevant laboratory findings during the index admission included elevated glucose, leukocytosis, hyponatremia, and hypokalemia. Persistent hyponatremia despite intravenous fluids prompted evaluation for syndrome of inappropriate antidiuretic hormone (SIADH), which was diagnosed on clinical and biochemical grounds; a serum antidiuretic hormone level was not obtained. Morning serum cortisol was mildly elevated at 25.1 mcg/dL with an evening value of 11.1 mcg/dL, indicating a normal daily pattern. Given the severity and recurrence of her hypertension, secondary causes were systematically evaluated and excluded. Renal function and renal ultrasonography were normal (baseline creatinine approximately 0.5 mg/dL), and echocardiography and electrocardiography were normal. Plasma‐free metanephrines and normetanephrine were normal, excluding pheochromocytoma and paraganglioma; thyroid function was normal; and aldosterone was within the reference range with an elevated renin, a pattern not suggestive of primary aldosteronism and consistent with volume contraction from recurrent vomiting. Cushing syndrome was also considered and excluded. No alternative secondary cause of her hypertension was identified (6).

**Figure 1:**
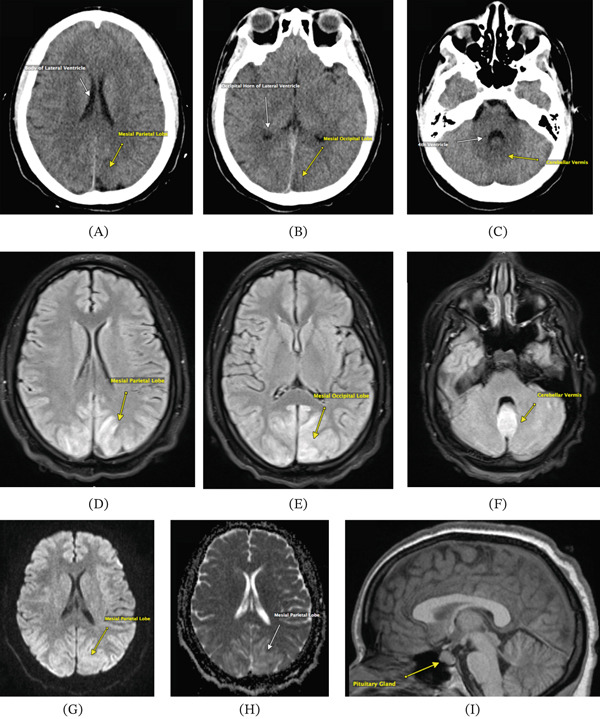
(A–C) Initial noncontrast axial CT of the head showed no abnormal cytotoxic or vasogenic edema. (D–F) Noncontrast axial MRI demonstrated symmetrical T2/FLAIR hyperintensities in the mesial parieto‐occipital lobes and cerebellar vermis bilaterally, consistent with vasogenic edema. (G–H) Diffusion‐weighted sequences showed increased DWI and ADC signal consistent with facilitated diffusion. (I) Noncontrast sagittal 3D T1 MPRAGE demonstrated nonspecific prominence of the pituitary gland without mass effect on the optic chiasm or suprasellar cistern.

Initial noncontrast CT of the head was unremarkable. The following day, noncontrast MRI of the brain demonstrated nonspecific prominence of the pituitary gland and symmetrical T2/FLAIR hyperintensities in the bilateral parieto‐occipital lobes and cerebellar vermis consistent with vasogenic edema. The constellation of the patient′s hypertensive state, clinical presentation, and imaging findings was compatible with PRES (Figure 1).

She was admitted to the pediatric intensive care unit for 2 weeks for management of PRES, including neurologic monitoring after the seizure, blood pressure control, and seizure prophylaxis and was discharged home following resolution of hypertension.

## 3. Discussion

This case describes a possible association between CVS and PRES, illustrating recurrent, predominantly hypertensive encephalopathy in a patient with CVS. PRES is a clinical and neuroimaging‐driven diagnosis that remains incompletely understood. It arises from a number of etiologies, including hematologic diseases, solid‐organ transplantation, oncological treatment, and renal disease (7–9). CVS itself is associated with autonomic dysregulation, predominantly sympathetic, and with hypertension in a subset of patients (3). A described phenotype, the Sato variant, couples periodic vomiting with hypertension and neuroendocrine findings including catecholamine discharge (4). In our patient, however, plasma metanephrines were normal, and cortisol was only mildly elevated with a normal daily pattern, so a Sato‐type mechanism is not biochemically supported. Her chronic, recurrent hypertension is the stronger link to PRES, and we therefore regard the relationship between her CVS and PRES as an association mediated primarily by hypertension, and possibly by CVS‐related autonomic dysregulation, rather than by a specific neuroendocrine variant. She has had two imaging‐confirmed episodes of PRES (in 2012 and the current presentation), the first with documented radiologic reversibility on follow‐up MRI, separated by additional hospitalizations for vomiting and hypertension that were not confirmed as PRES on neuroimaging. To our knowledge, recurrent PRES in a patient with CVS has not previously been reported. MRI with T2/FLAIR sequences has a high sensitivity in displaying lesions characteristic of PRES, including vasogenic edema predominantly in the parieto‐occipital regions with also atypical involvement of cerebellar vermis (10). These lesions often involve bilateral white‐matter regions but can also extend to gray matter in the supratentorial and infratentorial areas. Following contrast administration, there can be patchy variable enhancement in either a leptomeningeal or cortical pattern. GRE/SWI sequences can show susceptibility artifact related to microhemorrhages. Diffusion‐weighted sequences show increased DWI and ADC signal consistent with facilitated diffusion. Atypical patterns, including unilateral involvement or extensive edema with mass effect, involvement of basal ganglia and posterior fossa have also been observed in more severe cases.

Our patient exhibited a typical presentation of PRES, characterized by increased T2/FLAIR and ADC signals predominantly in the parieto‐occipital lobes and cerebellar vermis bilaterally, consistent with vasogenic edema. Her 2012 episode showed comparable parieto‐occipital involvement on MRI, with interval resolution on follow‐up imaging that confirmed reversibility. Additionally, the prominence of the pituitary gland on MRI, which may also reflect adolescent pituitary change, raises the possibility that vasogenic edema in regions involved in neuroendocrine regulation contributed to the concurrent SIADH.

Several other causes are worth considering. The index episode occurred during a period of sustained postoperative hypertension, and perioperative physiologic stress itself can precipitate hypertension and PRES; this mechanism cannot be excluded and may have acted alone or together with her underlying CVS‐related physiology. Renal, renovascular, and endocrine etiologies were assessed in our patient: Renal ultrasonography, echocardiography, thyroid studies, and aldosterone and renin were unremarkable, and pheochromocytoma, paraganglioma, and Cushing syndrome were excluded (6). Hypertensive encephalopathy and seizures can mimic PRES, and it is hard to be certain since the interval episodes were not imaged.

This report has several limitations. It describes a single patient, and the proposed link between CVS and PRES is hypothesis‐generating rather than confirmed. A temporal association between an active CVS attack and the hypertensive episode was not clearly documented; the contribution of perioperative stress cannot be separated from that of CVS, and only the 2012 and current episodes were confirmed with neuroimaging. The biochemical findings are nonspecific and were obtained in an acutely ill, postoperative patient, and normal metanephrines argue against the sustained catecholamine excess that characterizes the Sato variant. For these reasons, this case should be interpreted as an association rather than a proof of a direct causal mechanism.

## 4. Conclusion

This case describes recurrent, imaging‐confirmed PRES in an adolescent with long‐standing CVS and recurrent hypertension. The available data support hypertension as the immediate driver of PRES, whereas CVS‐related autonomic dysregulation may have contributed to her underlying susceptibility. A Sato‐type mechanism was not biochemically supported, and secondary renal, renovascular, and endocrine causes of hypertension were not identified. This case highlights the importance of careful blood pressure control, neuroimaging confirmation, and cautious interpretation of recurrent neurologic events in patients with CVS and severe episodic hypertension.

## Funding

No funding was received for this manuscript.

## Conflicts of Interest

The authors declare no conflicts of interest.

## Data Availability

Data sharing is not applicable to this article as no datasets were generated or analyzed during the current study.

## References

[1] Raucci U. , Borrelli O. , Di Nardo G. , Tambucci R. , Pavone P. , Salvatore S. , Baldassarre M. E. , Cordelli D. M. , Falsaperla R. , Felici E. , Ferilli M. A. N. , Grosso S. , Mallardo S. , Martinelli D. , Quitadamo P. , Pensabene L. , Romano C. , Savasta S. , Spalice A. , Strisciuglio C. , Suppiej A. , Valeriani M. , Zenzeri L. , Verrotti A. , Staiano A. , Villa M. P. , Ruggieri M. , Striano P. , and Parisi P. , Cyclic Vomiting Syndrome in Children, Frontiers in Neurology. (2020) 11, 583425, 10.3389/fneur.2020.583425, 33224097. 33224097 PMC7667239

[2] Hyams J. S. , Di Lorenzo C. , Saps M. , Shulman R. J. , Staiano A. , and van Tilburg M. , Childhood Functional Gastrointestinal Disorders: Child/Adolescent, Gastroenterology. (2016) 150, no. 6, 1456–1468.e2, 10.1053/j.gastro.2016.02.015, 27144632.

[3] Chelimsky T. C. and Chelimsky G. G. , Autonomic Abnormalities in Cyclic Vomiting Syndrome, Journal of Pediatric Gastroenterology and Nutrition. (2007) 44, no. 3, 326–330, 10.1097/MPG.0b013e31802bddb7, 17325552. 17325552

[4] Sato T. , Igarashi N. , Minami S. , Okabe T. , Hashimoto H. , Hasui M. , and Kato E. , Recurrent Attacks of Vomiting, Hypertension and Psychotic Depression: A Syndrome of Periodic Catecholamine and Prostaglandin Discharge, Acta Endocrinologica. (1988) 117, no. 2, 189–197, 10.1530/acta.0.1170189, 2837885. 2837885

[5] Fischer M. and Schmutzhard E. , Posterior Reversible Encephalopathy Syndrome, Journal of Neurology. (2017) 264, no. 8, 1608–1616, 10.1007/s00415-016-8377-8, 28054130. 28054130 PMC5533845

[6] Flynn J. T. , Kaelber D. C. , Baker-Smith C. M. , Blowey D. , Carroll A. E. , Daniels S. R. , de Ferranti S. D. , Dionne J. M. , Falkner B. , Flinn S. K. , Gidding S. S. , Goodwin C. , Leu M. G. , Powers M. E. , Rea C. , Samuels J. , Simasek M. , Thaker V. V. , Urbina E. M. , and SUBCOMMITTEE ON SCREENING AND MANAGEMENT OF HIGH BLOOD PRESSURE IN CHILDREN , Clinical Practice Guideline for Screening and Management of High Blood Pressure in Children and Adolescents, Pediatrics. (2017) 140, no. 3, e20171904, 10.1542/peds.2017-1904. 28827377

[7] Masetti R. , Cordelli D. M. , Zama D. , Vendemini F. , Biagi C. , Franzoni E. , and Pession A. , PRES in Children Undergoing Hematopoietic Stem Cell or Solid Organ Transplantation, Pediatrics. (2015) 135, no. 5, 890–901, 10.1542/peds.2014-2325. 25917987

[8] Musioł K. , Waz S. , Boroń M. , Kwiatek M. , Machnikowska-Sokołowska M. , Gruszczyńska K. , and Sobol-Milejska G. , PRES in the Course of Hemato-Oncological Treatment in Children, Child′s Nervous System. (2018) 34, no. 4, 691–699, 10.1007/s00381-017-3664-y, 29198072. PMC5856901 29198072

[9] Virojtriratana T. , Hongsawong N. , Wiwattanadittakul N. , Katanyuwong K. , Chartapisak W. , and Sanguansermsri C. , Comparison of Clinical Manifestations, Laboratory, Neuroimaging Findings, and Outcomes in Children With Posterior Reversible Encephalopathy Syndrome (PRES) in Children With and Without Renal Disease, Pediatric Neurology. (2022) 134, 37–44, 10.1016/j.pediatrneurol.2022.06.012, 35810661. 35810661

[10] Triplett J. D. , Kutlubaev M. A. , Kermode A. G. , and Hardy T. , Posterior Reversible Encephalopathy Syndrome (PRES): Diagnosis and Management, Practical Neurology. (2022) 22, no. 3, 183–189, 10.1136/practneurol-2021-003194, 35046115. 35046115

